# Arthroscopic Guided Synovial Biopsies

**DOI:** 10.3389/fmed.2020.604582

**Published:** 2021-02-11

**Authors:** Carl Kieran Orr, Elsa Vieira-Sousa, João Eurico Fonseca, Douglas Veale

**Affiliations:** ^1^Centre for Arthritis and Rheumatic Diseases, Saint Vincent's University Hospital, University College Dublin, Dublin, Ireland; ^2^Rheumatology Department, Hospital de Santa Maria, Centro Hospitalar Universitário Lisboa Norte, Lisbon Academic Medical Center, Lisbon, Portugal; ^3^Rheumatology Research Unit, Faculdade de Medicina, Instituto de Medicina Molecular João Lobo Antunes, Universidade de Lisboa, Lisbon, Portugal

**Keywords:** arthroscopy, synovial tissue, synovitis, inflammatory arthritis, synovial biopsy

## Abstract

Synovial tissue can be safely and reliably collected for research and clinical purposes using arthroscopy. This technique offers the obvious advantage of allowing direct visualization, and targeted biopsy of specific areas of interest within the joint, as well as for the collection of tissue which will include a lining layer. Much has been learnt by studying the synovium retrieved using this technique concerning the pathobiology of inflammatory arthritis. Furthermore, recent evidence suggests that the tissue retrieved may enable the identification of unique pathotypes that will allow for a precise approach to treatment selection in individual patients. Although ultrasound guided techniques for sampling synovial tissue have gained in popularity over the last decade, both methodologies are expected to compliment each other, each having unique benefits and drawbacks. We present here a detailed description of the arthroscopy technique reporting on our collective experience at two centers in Europe.

## Introduction

The synovium is the primary target tissue in inflammatory arthritis (IA), and it therefore follows that analysis of this tissue must yield important clues to advance our understanding of the underlying pathobiology of these heterogenous diseases. The field has rapidly expanded over the last three decades, and this has led to some very significant developments in unraveling the cellular and molecular networks underlying the development and perpetuation of IA (1–4). Putative targets have been identified by synovial tissue (ST) analysis (5). ST has been used in the evaluation of current and potential treatments, in both *in vivo* and *ex vivo* settings (6, 7). Recent evidence suggests that it may be possible to stratify patients with rheumatoid arthritis (RA) on the basis of histopathology and transcriptomic analysis, into groups with differing underlying pathobiology, and with differential responses to therapies (8–10). These developments have depended on the ability to reliably retrieve ST, in a safe manner, and in such a way as to be well-tolerated by patients. Although much data has been published on tissue retrieved at arthroplasty, the suitability and applicability of these findings to IA at much earlier timepoints in the disease course remains unclear. Therefore, arthroscopy was adopted by Rheumatologists to allow ST sampling at varying points in the disease course and has long been the favored technique historically. This technique has the advantage of providing direct intra-articular visualization of synovium as well as a therapeutic joint lavage. Both synovial membrane proliferation and vascularization patterns have been described. Certain vascular patterns, although not diagnostic, can be suggestive of some subtypes of inflammatory arthritis (straight versus tortuous pattern) which can be of particular interest in early and undifferentiated arthritis. Furthermore, crystal deposits, cartilage damage, chondromas and other intra-articular pathologies can be identified contributing for the differential diagnosis of synovitis (11, 12). Sonographically guided techniques have been developed and refined more recently, and it is likely that these techniques will complement ST retrieved under direct arthroscopic guidance. In this review, we describe the general aspects of the technique of arthroscopic guided synovial biopsies (AGSB) of the knee joint under local anesthetic, as performed in two European Rheumatology Centers: St. Vincent's University Hospital, Dublin and Hospital de Santa Maria, Lisbon.

## Patient Recruitment and Indication for AGSB

The authors of this review (CO, EV-S, DV) have experience performing AGSB for patients recruited at the Inflammatory Arthritis Clinics at St. Vincent's University Hospital in Dublin, and from the dedicated Mini-arthroscopy Clinic at Hospital de Santa Maria in Lisbon. Patients are referred either with undifferentiated arthritis or an arthritis flare of an established IA for potential sampling, for diagnostic and/or therapeutic purposes. The specific organization of these clinics allows for enrichment for recruitment, and all medical staff at each center are made aware of the clinical benefits and research programs, and they contribute to patient recruitment. These arthroscopy clinics are focused solely on knee arthritis, and patients with swollen and painful knees are referred.

Knee arthroscopies are performed on patients with a wide range of diagnoses or potential diagnoses: most commonly this is rheumatoid arthritis (RA), undifferentiated arthritis, or spondylarthritis. Furthermore, we have found that most patients are willing to consent to a second arthroscopy and are often less apprehensive about this, having already experienced the procedure (13). This commonly occurs when a treatment change is indicated following arthroscopy, in which circumstance patients are invited to return for follow up ST sampling 12 weeks later.

## Ethics and Consent

The patient is given written information in the form of a Patient Information Leaflet (PIL) that contains relevant information regarding the procedure, the potential risks and instructions post-procedure. For example, patients are instructed not to drive immediately after the arthroscopy, and to rest for the first 48 h. The consent form and the PIL are provided to the patient at the time of recruitment. Local Ethics Committees approve all studies, including the procedure itself where this is conducted exclusively for research purposed. Patient confidentiality is a priority and no identifiable data is retained. If agreeable, patients are scheduled to attend for arthroscopy at the next available slot. We endeavor to ensure that this is within 2 weeks, in order to provide timely access to diagnosis, therapeutic benefit and treatment initiation or change.

On the morning of arthroscopy, patients are requested to bring their PIL/informed consent and there is further discussion with the physician performing the procedure, and an opportunity for any questions to be answered. The patients are invited to consent to the procedure itself, the collection and retention of synovial tissue and blood for research purposes, as well as the collection and anonymous storage of particular demographic data and disease features.

## Patient Data

Relevant demographic and disease specific data including ongoing medication, disease activity (e.g., tender and swollen joint counts, patient global health evaluation) as well as validated patient reported outcomes such as the SF-36 and HAQ, as well as the indication for the procedure, are captured. On the morning of the procedure, patients are told to have a light breakfast, and on arrival, the completed forms are checked, and a comprehensive clinical examination is performed and recorded, including various disease activity measurements.

## Pre-procedure Assessment

A detailed patient's medical history is collected and a plain film radiograph of the knee is requested (if not available) as well as laboratorial evaluation including coagulation parameters. For example, antiaggregant such as clopidogrel and direct oral anti-coagulants or warfarin, are contraindicated unless these can safely be discontinued for an appropriate time before the procedure or occasionally replaced by low molecular weight heparin.

## Procedure

Arthroscopies are performed in a dedicated, facility, within the hospital's Clinical Research Center (Dublin) and within the Rheumatolgy Technical Procedures Unit (Lisbon). The unit comprises an anteroom and a procedure room. Patient's change into hospital gowns in the anteroom and usually an intravenous cannula is sited. Cannulation facilitates phlebotomy, for both research bloods and the hospital laboratory to process relevant hematological and biochemical indices. The knee that is to be the site of sampling is marked with a skin marker.

We start by switching the arthroscopy tower on, and by ensuring that both the camera and the light source are working. Patient's hospital identification and knee laterality is inserted for image capture. At both centers, Karl Storz, Germany, equipment are used. In Dublin a 2.7 mm needle arthroscope is used and in Lisbon a 2.7 mm Hopkins II, 30° telescope. The operator scrubs and gowns using standard techniques described elsewhere (14). A theater nurse assists the operator before and throughout the procedure but does not scrub, respecting well-defined aseptic areas.

**Figure 1:**
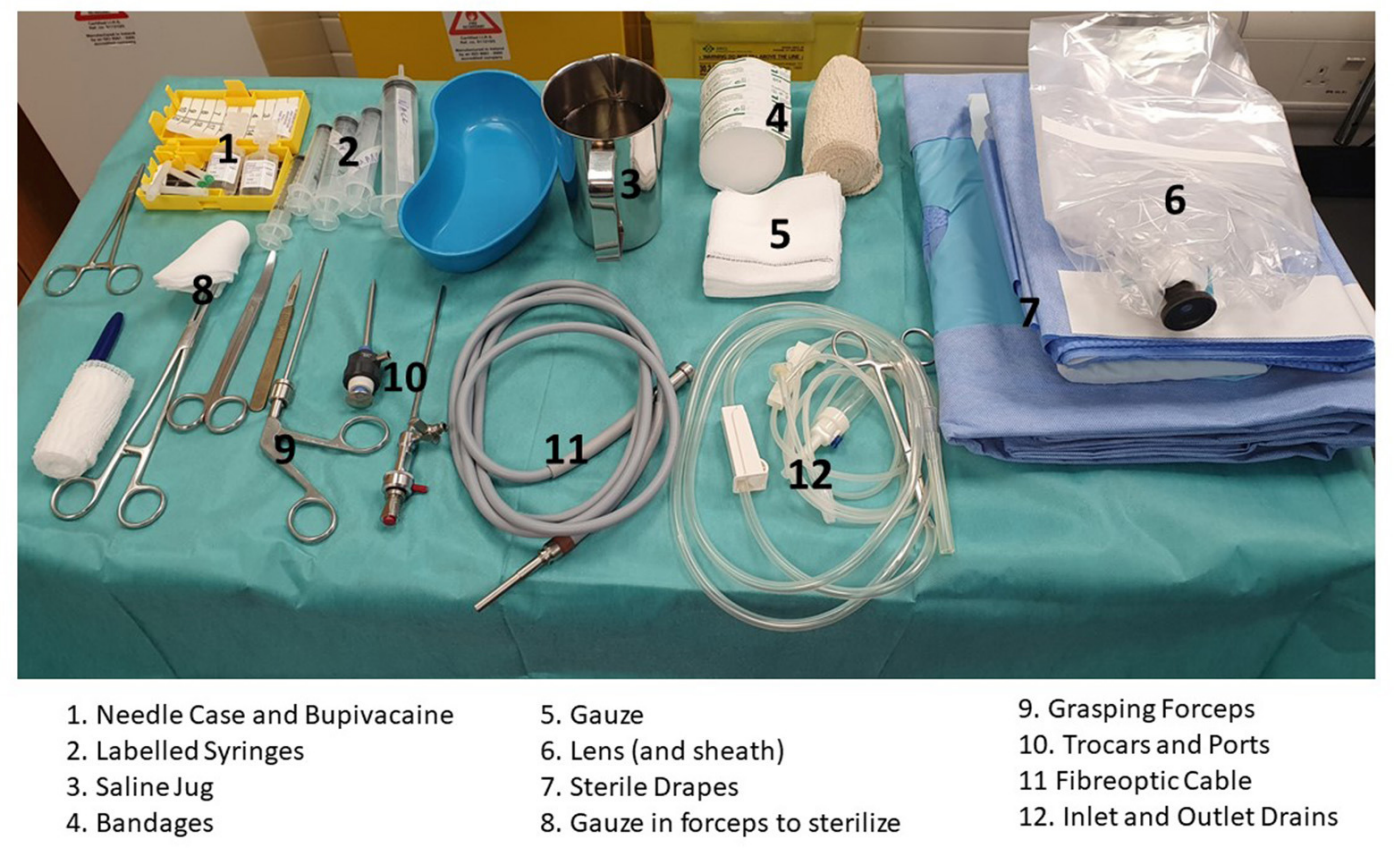
Arthroscopy equipment.

All surgical instruments and the Karl Storz hardware kit which contain arthroscopy telescope, trocars, forceps, are assembled in sterile packs and opened on a sterile draped table. In addition, sterile gauzes, syringes, needles, infusion system, sterile bowls and sterile drapes are opened by the theater nurse, received by the operator, and placed on the table. Chlorhexidine and sterile water are poured by the nurse into the sterile bowls on the table. A 20 ml syringe is filled with lignocaine 2%, and another 20 ml syringe filled with bupivacaine 0.5% (Dublin). The local anesthetic products and expiry dates are checked by both the nurse and the operator. Figure 1 depicts the prepared equipment assembled on a sterile trolley.

Before proceeding any further, a deliberate pause is undertaken to complete a reviewed “WHO Surgical Safety Checklist” for this procedure. Amongst the most important items on the list are a check that we have the correct patient, that the correct knee has been labeled, and that equipment has been arranged for this knee to be biopsied, as well as a final check to confirm the consent form has been completed, and that the patient is not on anti-coagulants/anti-aggregants, and an allergy check. Throughout the procedure we pay careful attention to the patient's comfort and explain each step as we proceed.

A disposable sterile sheet is placed under the lower limb and the leg is raised to 45°, with the assistance of the nurse who holds the patient's heel up. Sterile gauze is held in a pair of forceps and soaked in Chlorexidine. The knee is sterilized by swabbing in ever widening circles from the lateral aspect of the knee, where the two small incisions will be made. The area from mid-thigh to mid-calf is disinfected circumferentially.

In Dublin, a bespoke sterile sleeve closed at one end is placed over the foot and extended the length of the leg with care taken not to contaminate the cleaned field. The leg is next inserted through the fenestration in a sterile drape and the drape advanced to the hip. The two corners of the drape closest to the cephalic end are risen and attached to drip stands either side of the bed. This occludes the patient from inadvertently touching the sterile field. A sterile gauze bandage is applied circumferentially around ankle (which is covered by the sleeve). A window large enough to access the antero-lateral aspect of the knee is cut out of the sterile sleeve using a pair of scissors, taking care not to injure the skin. A similar setup with minor differences is performed in Lisbon.

The anatomical landmarks must now be identified. The knee is placed in 30–45 degrees flexion. Following palpation, a tissue marker can be used to delineate the lateral border of the infrapatellar tendon, the infero-lateral aspect of the patella, the anterolateral border of the tibial plateau, and the infero-lateral surface of the lateral epicondyle. The center of these markings is the site of entry for infero-lateral port. The landmark for the supero-lateral port is 1 cm above and 1 cm lateral to the supero-lateral aspect of the patella, the site used commonly for intra-articular injections. The joint capsule, soft tissues and skin is infiltrated with 10 ml of lignocaine 2%, for what will become the superior-lateral port site and the inferior-lateral port site. About 2–5 min is given to allow for this to take effect. Arthrocentesis is performed using a 21G needle, attached to an empty 10/20ml syringe through the site of the prospective supero-lateral port and synovial fluid is drained until no further fluid can be removed. Obtaining synovial fluid at this point gives high assurance that the tip of the needle is indeed in the joint cavity. Any synovial fluid removed is carefully placed on the sterile drape covered table. If no fluid can be removed, special care must be taken in the next steps.

The needle is left *in situ* while the syringe is detached. The 20 ml bupivacaine containing syringe is attached to the needle. There should be little or no resistance to the plunger advancing. If the operator encounters resistance, especially where no synovial fluid was obtained, the needle may be misplaced and may require adjustment. After placement of the bupivacaine into the joint cavity, the needle is left *in situ* and a further 2–5 min is given to allow the bupivacaine to take effect.

A sterile sleeve covers the camera wire and camera head, and the sterilized wire light source is attached to the camera. An infusion system attached to a saline solution is also connected to the telescope trocar. The white balance is ensured by placing a gauze in front of the lens.

In Dublin in the next step, the empty syringe is detached from the needle and the 50 ml syringe containing sterile water is attached to the needle. The contents of the syringe are placed into the joint until resistance is felt. Typically, a joint will accommodate up to 60 ml more fluid.

With the knee in a 30–45 degree flexed position, a scalpel is used to make a 1 cm incision in the infra-lateral patella space previously delineated, extending to the joint capsule. The trocar is introduced through the incision using blunt dissection into the joint cavity. It is imperative not to force the trocar if resistance is met. Once *in situ*, the trocar is removed, leaving the port in position, and the rigid camera is inserted through the infero-lateral port.

**Figure 2:**
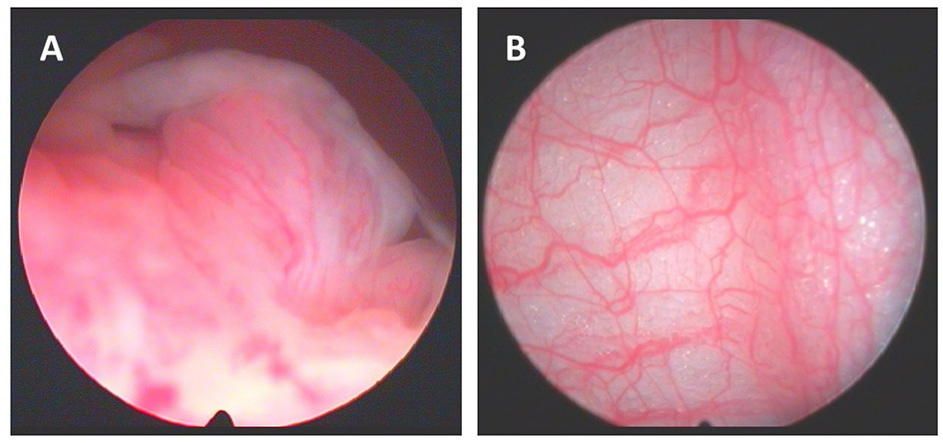
**(A)** Macroscopic aspects of synovitis with villi formation and **(B)** synovium vascularization.

The cavity of the knee joint can now be inspected. It is important to make certain that the arthroscopy is being video recorded and that photographs are taken. We inspect all visually accessible areas and arbitrarily divide the joint into discrete compartments, as occasionally synovitis can be quite focal. We record synovitis and vascularity scores as appraised by the operator on visual analog scales ranging from 0 to 100 mm (Dublin) or a 0 to 3 severity scale (Lisbon). There have been attempts to develop a reliable scoring system for synovitis observed at arthroscopy, but until recently the numbers studied were small and no system has been validated (15). Our data suggests that there is a correlation between macroscopic synovitis scores and C reactive protein, histological inflammation and the development of erosions (16). More recently, a comprehensive scoring system has been proposed called the “Macro-score,” and has been shown to exhibit excellent inter- and intra-rater variation (17). We also record the pattern of vascularity which has been shown to differ between, for example RA and psoriatic arthritis (11, 18). Figure 2 depicts exemplar images for synovitis and vascularity. Other notable findings are also recorded, such as synovial crystal deposits, tophi or any other intra-articular pathologies. The under-surface of the patella is inspected, and any chondropathy observed is recorded.

To establish the second port required to allow AGSB, the camera is placed such that it points toward to area where the superior-lateral port will be positioned. This can be found by using a finger to exert pressure over the site, and the operator can see the depression made by this action within the joint on screen. Once again, a scalpel is used to make a 1 cm incision extending to the joint capsule and then blunt dissection using a trocar allows the second port be sited.

**Figure 3:**
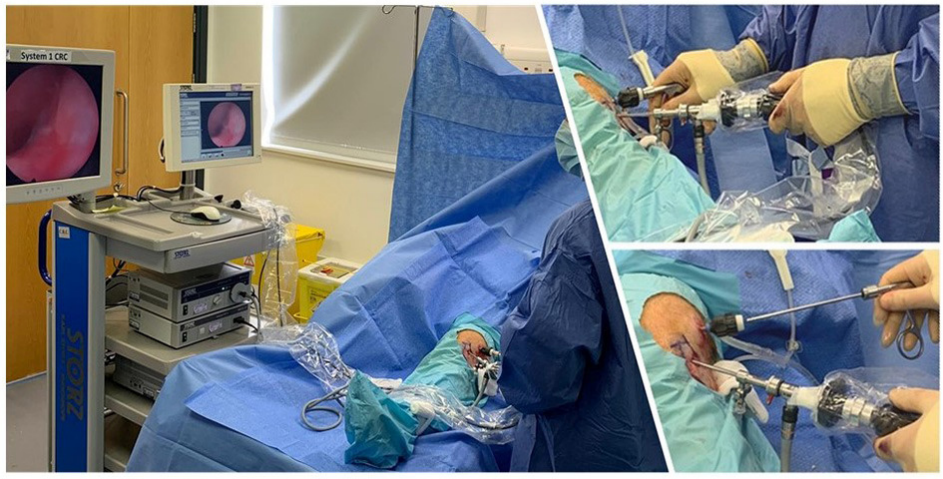
Ilustrative images of arthroscopy procedure.

Sterile saline *via* a drip is connected to the camera port, and a drain connected to the grasper port with tubing to a basin or collection bag. A rigid grasper is inserted through the supero-lateral port and synovial biopsies can be collected under direct visualization. Figure 3 illustrates the positioning of the ports and their relationship to the other equipment discussed above. For time optimization, when diffuse synovitis is present biopsies can be easily performed without direct visualization. The biopsies are placed on saline soaked gauze. When all biopsies have been taken, they are immediately collected to minimize delay in processing.

We check for any bleeding after biopsy collection and complete an effective joint lavage-usually approximately 1,500 ml thought the procedure. The superolateral trocar is removed after draining all the fluid from the joint.

Whenever indicated, intra-articular corticosteroids, are administrated. This is followed by the administration of 8 ml of bupivacaine is intra-articular to allow a sustained anesthetic benefit (Lisbon). The infrapatellar port (and camera) is removed. The two port sites are wiped clean, and either paper stitches or a single stich at each portal are applied to each incision. We next apply a small square dressing to each incision and apply a waterproof patch. Now the sterile drapes and sleeve are removed, and the knee is wrapped firstly with a cotton wool bandage (Dublin) and then with a crepe bandage (Dublin and Lisbon).

## Post-procedure Assessment

A procedure note is recorded which includes synovitis and vascularity scores, blood vessel pattern, chondropathy, crystal deposits if present, and whether intra-articular steroids were administered as well as the amount of saline used during the arthroscopy. We have developed a proforma to make this faster and to standardize the procedure notes.

The patient is given written information concerning post procedural care. They are told to remove the crepe and cotton wool bandage after 12–24 h, a waterproof cover is useful to keep the knee dry for 3 days, and to remove the dressing and paper (Dublin) or surgical stitches (Lisbon) in 7 days. They are told to rest, and not to drive for 48 h. If they have persistent swelling we ask them to apply ice. Patients are given emergency contact details for the Rheumatology department, and are given an outpatient appointment for 1 week or 2 weeks' time, when the wounds are inspected, the results from synovial fluid/synovial membrane verified and any indicated change to treatment is implemented.

## Safety and Tolerability

Overall safety and tolerability of AGSB in the hands of rheumatologists is reflected in several publications including those from our groups in Dublin and from integrated data from Lisbon in an international study recently published by Just et al. (13, 19). We quote our patients for adverse events related to the procedure such as persistent swelling, haemarthrosis (0.2%), DVT (0.9%), and septic arthritis (<0.1%).

## Diagnostic and Therapeutic Benefits

AGSB performed by Rheumatologists is associated with diagnostic and therapeutic benefits. Collecting the tissue allows for appropriate histologic and microbiologic evaluation of the synovial membrane, but additionally AGSB allows for the direct visualization of intra-articular space as well as facilitating an efficacious joint lavage. Additionally, intra-articular corticosteroids and/or anesthetics can be administered with symptomatic relief. Remarkably, 66.9% (91/136) of our patients felt improvement in their knee symptoms within 2 weeks of arthroscopy (20). Some factors which may explain this include the removal of inflammatory synovial fluid, the knee lavage itself, and the intraarticular injection of corticosteroids in a minority of the patients surveyed. Although few comparative studies have been published, arthroscopic joint lavage plus intra-articular corticosteroids injection is superior to intra-articular injection of corticosteroid alone following joint aspiration in a randomized clinical trial (21).

## Challenges

It is widely accepted that AGSB requires technical skills and dedicated training for the operator, which is currently restricted to a small number of academic centers. In addition to this challenge, international guidelines standardizing AGSB as performed by rheumatologists, are scarce (22). As noted by Smits et al., it is certain that ongoing performance of the procedure is important in maintaining skills, which can be appraised by examining biopsy yield and quality, as well as safety record, but it is not known how many supervised procedures are required to attain competency, and how frequently procedures should be performed to maintain this competency, and these factors might be operator dependent (23).

Humby et al. has reviewed the pros and cons of the various methodologies for biopsying synovium (24). The preferred method will likely depend on the clinical or research question being addressed, and in particular, whether lining layer is essential, and how much tissue is required. The obvious benefit of arthroscopy for ST collection is the reliable, relatively large quantity of material retrieved, and the ability to target of areas of most significant synovitis within the joint under direct visualization, as well as reliably collecting lining layer. One limitation of AGSB is its general restriction to large joints. However, there is some evidence to suggest that tissue retrieved from an inflamed knee joint is similar to that obtained contemporaneously from an inflamed wrist or metacarpophalangeal joint (25). Furthermore, in subjects with clinically evident disease manifest in small joints, similar histological abnormalities have been recorded in apparently clinically uninvolved knee joints (26, 27). Taken together, these findings would suggest that AGSB of a large joint, should be representative tissue for studying IA from a histologic perspective. Joint specific synovial fibroblast phenotypes have also been described owing to anatomical transcriptional diversity, and this may have implications for the wider applicability of sampling from any given single joint (28). Of interest, recent evidence suggests that DNA methylation and transcriptome signatures in RA fibroblast-like synoviocytes can vary between knees and hips for example, but the clinical implications for diagnostic or therapeutic decisions in clinical practice from this data is still limited (29).

Additionally, non-swollen joints can also be biopsied with success such as those from patients with osteoarthritis or after successful treatment inflammatory arthritis.

## Conclusion

AGSB as performed by rheumatologists is a safe and reliable technique for sampling synovial tissue that is most suited to large joints. It has been the preferred “gold-standard” method, and for the last decade the cornerstone for the development of newer synovial tissue biopsy techniques, namely sonographically guided. It is hoped that the addition of these tools may broaden the accessibility of using synovial biopsies in research and clinical settings in the Rheumatology field. Arthroscopy will however undoubtedly remain an important tool in investigating IA with complementary therapeutic benefits, and specifically identifying synovial biomarkers that will allow the page to be turned toward precision medicine for our patients with heterogenous IA.

## Author Contributions

CO, EV-S, and DV have extensive experience in performing this procedure, and have contributed to the collection of data relevant to this review. JEF has significantly contributed to arthroscopy implementation in Lisbon. All authors have contributed to writing and editing the manuscript.

## Conflict of Interest

The authors declare that the research was conducted in the absence of any commercial or financial relationships that could be construed as a potential conflict of interest.

## References

[1] Orr C Vieira-Sousa E Boyle DL Buch MH Buckley CD Cañete JD. Synovial tissue research: a state-of-the-art review. Nat Rev Rheumatol. (2017) 13:630. 10.1038/nrrheum.2017.161 28935945

[2] Dorris ER Linehan E Trenkmann M Veale DJ Fearon U Wilson AG. Association of the rheumatoid arthritis severity variant rs26232 with the invasive activity of synovial fibroblasts. Cells. (2019) 8:1300. 10.3390/cells8101300 31652652 PMC6829881

[3] Wade SM Canavan M McGarry T Low C WadeS C Mullan RH. Association of synovial tissue polyfunctional T-cells with DAPSA in psoriatic arthritis. Ann Rheum Dis. (2018) 4:350–4. 10.1136/annrheumdis-2018-214138 30626658 PMC6390025

[4] Harre U Georgess D Bang H Bozec A Axmann R Ossipova E. Induction of osteoclastogenesis and bone loss by human autoantibodies against citrullinated vimentin. J Clin Invest. (2012) 122:1791–1. 10.1172/JCI60975 22505457 PMC3336988

[5] Harty LC Gerlag DM Pitzalis C Pitzalis C Veale DJ Tak PP. Synovial tissue analysis for the discovery of diagnostic and prognostic biomarkers in patients with early arthritis. J Rheumatol. (2011) 38:2068–72. 10.3899/jrheum.110426 21885519

[6] Firestein GS Paine MM Boyle DL. Mechanisms of methotrexate action in rheumatoid arthritis. Arthritis Rheum. (1994) 37:193–200. 8129774 10.1002/art.1780370207

[7] Tak PP Van Der Lubbe PA Cauli A Daha MR Smeets TJ Kluin PM. Reduction of synovial inflammation after anti-CD4 monoclonal antibody treatment in early rheumatoid arthritis. Arthritis Rheum. (1995) 38:1457–65. 7575695 10.1002/art.1780381012

[8] Dennis G Holweg CTJ Kummerfeld SK Choy DF Setiadi AF Hackney JA. Synovial phenotypes in rheumatoid arthritis correlate with response to biologic therapeutics. Arthritis Res Ther. (2014) 16. 10.1186/ar4555 25167216 PMC4060385

[9] Humby F Lewis M Ramamoorthi N Hackney JA Barnes MR Bombardieri M. Synovial cellular and molecular signatures stratify clinical response to csDMARD therapy and predict radiographic progression in early rheumatoid arthritis patients. Ann Rheum Dis. (2019) 78:761–72. 10.1136/annrheumdis-2018-214539 30878974 PMC6579551

[10] Lewis MJ Barnes MR Blighe K Goldmann K Rana S Hackney JA. Molecular portraits of early rheumatoid arthritis identify clinical and treatment response phenotypes. Cell Rep. (2019) 28:2455–70.e5. 10.1016/j.celrep.2019.07.091 31461658 PMC6718830

[11] Reece RJ Canete JD Parsons WJ Emery P Veale DJ. Distinct vascular patterns of early synovitis in psoriatic, reactive, and rheumatoid arthritis. Arthritis Rheum. (1999) 42:1481–4. 10403277 10.1002/1529-0131(199907)42:7<1481::AID-ANR23>3.0.CO;2-E

[12] Cañete JD Rodríguez JR Salvador G Gómez-Centeno A Muñoz-Gómez J Sanmartí R. Diagnostic usefulness of synovial vascular morphology in chronic arthritis. A systematic survey of 100 cases. Semin Arthritis Rheum. (2003) 32:378–7. 10.1053/sarh.2002.50004 12833246

[13] Just SA Humby F Lindegaard H de Bellefon LM Durez P Vieira-Sousa E. Patient-reported outcomes and safety in patients undergoing synovial biopsy: Comparison of ultrasound-guided needle biopsy, ultrasound-guided portal and forceps and arthroscopic-guided synovial biopsy techniques in five centres across Europe. RMD Open. (2018) 4:1–10. 10.1136/rmdopen-2018-000799 30488001 PMC6241983

[14] WHO. Best Practice Protocols Clinical Procedures Safety. World Health Organization. (2007). http://www.who.int/surgery/publications/www.who.int/surgery/publications/BestPracticeProtocolsCPSafety07.pdf (accessed April 1, 2020).

[15] Lindblad S Hedfors E. Intraarticular variation in synovitis. Local macroscopic and microscopic signs of inflammatory activity are significantly correlated. Arthritis Rheum. (1985) 28:977–86. 10.1002/art.1780280904 2931087

[16] Orr C Mcgarry T Young F Linehan E Fearon U Veale D. SAT0040 macroscopic scores of synovitis at knee arthroscopy correlate well with CRP, inflammatory histology findings, and can predict later erosive disease on hands and feet plain film radiographs. Ann Rheum Dis. (2015) 74:662.4–3. 10.1136/annrheumdis-2015-eular.5316

[17] af Klint E Catrina AI Matt P Neregråd P Lampa J Ulfgren A-K. Evaluation of arthroscopy and macroscopic scoring. Arthritis Res Ther. (2009) 11:1–13. 10.1186/ar2714 19490631 PMC2714131

[18] Fearon U Griosios K Fraser A Reece R Emery P Jones PF. Angiopoietins, growth factors, and vascular morphology in early arthritis. J Rheumatol. (2003) 30:260–8. 12563678

[19] Kane D Veale DJ FitzGerald O Reece R. Survey of arthroscopy performed by rheumatologists. Rheumatol. (2002) 41:210–5. 10.1093/rheumatology/41.2.210 11886972

[20] Orr C MacMullan P Gallagher P Murray M O'Neill M Veale DJ. Knee arthroscopy in an international training centre: an audit of safety and impact on work days. In: American College of Rheumatology Annual Congress (2014).

[21] van Oosterhout M Sont JK Bajema IM Breedveld FC van Laar JM. Comparison of efficacy of arthroscopic lavage plus administration of corticosteroids, arthroscopic lavage plus administration of placebo, and joint aspiration plus administration of corticosteroids in arthritis of the knee: a randomized controlled trial. Arthritis Rheum. (2006) 55:964–70. 10.1002/art.22340 17139644

[22] van de Sande MGH Gerlag DM Lodde BM van Baarsen LGM Alivernini S Codullo V. Evaluating antirheumatic treatments using synovial biopsy: a recommendation for standardisation to be used in clinical trials. Ann Rheum Dis. (2011) 70:423–7. 10.1136/ard.2010.139550 21109518

[23] Smits M van de Groes S Thurlings RM. Synovial tissue biopsy collection by rheumatologists: ready for clinical implementation? Front Med. (2019) 138:10–12. 10.3389/fmed.2019.00138 31281817 PMC6595539

[24] Humby FC. Synovial tissue sampling in rheumatological practice-past developments and future perspectives. Front Med. (2019) 4:4–8. 10.3389/fmed.2019.00004 30761301 PMC6361834

[25] Kraan MC Reece RJ Smeets TJM Veale DJ Emery P Tak PP. Comparison of synovial tissues from the knee joints and the small joints of rheumatoid arthritis patients: Implications for pathogenesis and evaluation of treatment. Arthritis Rheum. (2002) 46:2034–8. 10.1002/art.10556 12209505

[26] Kraan MC Versendaal H Jonker M Bresnihan B Post WJ Hart BA. Asymptomatic synovitis precedes clinically manifest arthritis. Arthritis Rheum. (1998) 41:1481–8. 10.1002/1529-0131(199808)41:8<1481::aid-art19>3.0.co;2-o 9704648

[27] Soden M Rooney M Cullen A Whelan A Feighery C Bresnihan B. Immunohistological features in the synovium obtained from clinically uninvolved knee joints of patients with rheumatoid arthritis. Br J Rheumatol. (1989) 28:287–92. 2787183 10.1093/rheumatology/28.4.287

[28] Frank-Bertoncelj M Trenkmann M Klein K Karouzakis E Rehrauer H Bratus A. Epigenetically-driven anatomical diversity of synovial fibroblasts guides joint-specific fibroblast functions. Nat Commun. (2017) 8:14852. 10.1038/ncomms14852 28332497 PMC5376654

[29] Ai R Hammaker D Boyle DL Morgan R Walsh AM Fan S. Joint-specific DNA methylation and transcriptome signatures in rheumatoid arthritis identify distinct pathogenic processes. Nat Commun. (2016) 7:11849. 10.1038/ncomms11849 27282753 PMC4906396

